# Associations Between the Food Environment and Food Insecurity on Fruit, Vegetable, and Nutrient Intake, and Body Mass Index, Among Urban-Dwelling Latina Breast Cancer Survivors Participating in the *¡Mi Vida Saludable*! Trial

**DOI:** 10.3390/nu17243950

**Published:** 2025-12-17

**Authors:** Zachary O. Kadro, Eileen Rillamas-Sun, Blake O. Langley, Allison Meisner, Isobel Contento, Pamela A. Koch, Ann Ogden Gaffney, Dawn L. Hershman, Heather Greenlee

**Affiliations:** 1 Department of Family Medicine and Community Health, Oakland University William Beaumont School of Medicine, Rochester, MI 48309, USA; 2 Integrative Medicine, Corewell Health, William Beaumont University Hospital, Royal Oak, MI 48073, USA; 3 Integrative Medicine Program, Fred Hutchinson Cancer Center, Seattle, WA 98109, USA; hgreenlee@fredhutch.org; 4 Cancer Prevention Program, Fred Hutchinson Cancer Center, Seattle, WA 98109, USAblangley@fredhutch.org (B.O.L.); ameisner@fredhutch.org (A.M.); 5 Department of Hematology Oncology, University of Washington School of Medicine, Seattle, WA 98195, USA; 6 Program in Nutrition, Department of Health Studies and Applied Educational Psychology, Teachers College Columbia University, New York, NY 10027, USA; irc6@columbia.edu (I.C.);; 7 Cook for Your Life, New York, NY 10024, USA; 8 Department of Medicine, Columbia University Medical Center, New York, NY 10032, USA; dlh23@cumc.columbia.edu; 9 Herbert Irving Comprehensive Cancer Center, Columbia University Medical Center, New York, NY 10032, USA

**Keywords:** food insecurity, food environment, fruit and vegetable intake, Latina, breast cancer survivors

## Abstract

**Background**: Socioeconomic disparities may drive cancer inequities in Hispanic/Latino populations. We examined associations of perceived access to healthy foods (AHF) and food insecurity (FI) with diet and body mass index (BMI) changes in Latina breast cancer (BC) survivors. **Methods**: Latina BC survivors in a 12-month intervention trial aiming to increase fruit/vegetable intake and physical activity were analyzed. AHF was from a modified, validated neighborhood environment scale and dichotomized (low–medium vs. high). FI was defined as eating less and/or going hungry due to a lack of money. AHF and FI surveys were self-reported. Outcomes included dietary intake, diet quality, and BMI. Fruit/vegetable intake was log-transformed. Relationships between AHF and FI and changes in diet and BMI were evaluated using generalized estimating equations. **Results**: Of women with AHF data (*n* = 86), 58% reported low–medium access and 42% reported high access. Fruit/vegetable (FV) intake declined overall from baseline to 12 months, with greater reductions among low–medium AHF women (−32%, 95% CI: −51%, −7%) compared with high AHF women (−17%, 95% CI: −40%, +13%). Statistically significant 12-month decreases in total calories, carbohydrates, sugars, and fat occurred in low–medium AHF women but not high AHF women, and changes in total energy density, carbohydrates, sugars, and BMI at 12 months were statistically significantly different between women with low–medium AHF and women with high AHF, *p* ≤ 0.05. Among 157 women, 23% reported FI. Reductions in fruit/vegetable intake were larger in women with FI (−39%, 95% CI: −57%, −14%) than in women without FI (−10% reductions, 95% CI: −25%, +8%) and between-group differences were significant at both 6 and 12 months, *p* ≤ 0.05. Most diet measures decreased for both FI and non-FI women, with greater decreases among those with FI. **Conclusions**: Latina BC survivors with FI or perceived limited AHF experienced greater declines in indicators of healthy diets including FV intake. Future interventions should integrate strategies to measure AHF and FI to address disparate access to healthy food options.

## 1. Introduction

For Hispanic/Latina women in the United States (US), breast cancer is the most commonly occurring cancer and is the leading cause of cancer death [1]. While Hispanics/Latinas living in the US have a lower lifetime probability of developing and dying of breast cancer compared to non-Hispanic Whites, they are more likely to be diagnosed with more advanced disease and have a lower 5-year survival rate [1,2]. Many reasons for the disparity in breast cancer survival between Hispanic/Latina and non-Hispanic White women have been suggested, including differences in the timing of screening, how the cancer is treated and managed, access to care and health insurance, behavioral risk factors, and socioeconomic and cultural influences [1,3,4,5,6,7,8,9].

Socioeconomic influences represent a broad range of attributes. Studies examining the role of socioeconomic factors on ethnic disparities in breast cancer development and mortality have focused on insurance status [5,9], income and poverty [6,10,11], and neighborhood characteristics [4,9,12], such as census-level descriptors like urbanization, rurality, and economic status in the composition of residents. These studies highlight that ethnic disparities in breast cancer outcomes are multifactorial and nuanced. For example, Kish et al. examined cancer survival by quintiles of neighborhood socioeconomic status (SES) [4]. They found no differences in breast cancer mortality between Hispanics/Latinas and non-Hispanic Whites living in the lowest quintile of neighborhood SES, but a 20% higher risk for Hispanic women relative to non-Hispanic White women living in the highest quintile of neighborhood SES [4].

In addition to SES-related risk factors, modifiable health behaviors might contribute to poorer breast cancer outcomes for Hispanic/Latina women with breast cancer. Studies have shown that Hispanic/Latina breast cancer survivors do not tend to engage in healthy lifestyle behaviors, such as consuming high-quality diets with fruits and vegetables and engaging in moderate-to-vigorous physical activity (MVPA) [1,13,14,15]. This is important because specific nutrients present in fruits and vegetables are protective against breast cancer. These nutrients include dietary fiber, vitamins C, D, E, folate and B6, carotenoids (including alpha-carotene, beta-carotene, lutein, and zeaxanthin) found in yellow and orange vegetables (e.g., carrots and peppers), polyphenols and flavonoids found in citrus, apples and berries, and glucosinolates found in cruciferous vegetables (e.g., broccoli and cauliflower) that are metabolized into sulforaphane and indole-3 carbinol/3,3’-diindolylmethane (I3C/DIM). Through a variety of mechanisms, these nutrients exert antioxidant and anti-inflammatory effects, modulate estrogen metabolism, and induce apoptosis [16,17,18]. The American Cancer Society recommends that breast cancer survivors consume at least five servings of fruits and vegetables daily and participate in MVPA for at least 150 min per week to reduce the risk of recurrence and mortality [19]. Yet, the extent to which food insecurity and the food environment affect the capacity of breast cancer survivors to consume a high-quality diet has not been well-examined. Although numerous studies have reported the associations of neighborhood SES and recurrence, mortality, and outcomes in breast cancer survivors, associations with characteristics of the neighborhood environment have been lacking.

**Figure 1:**
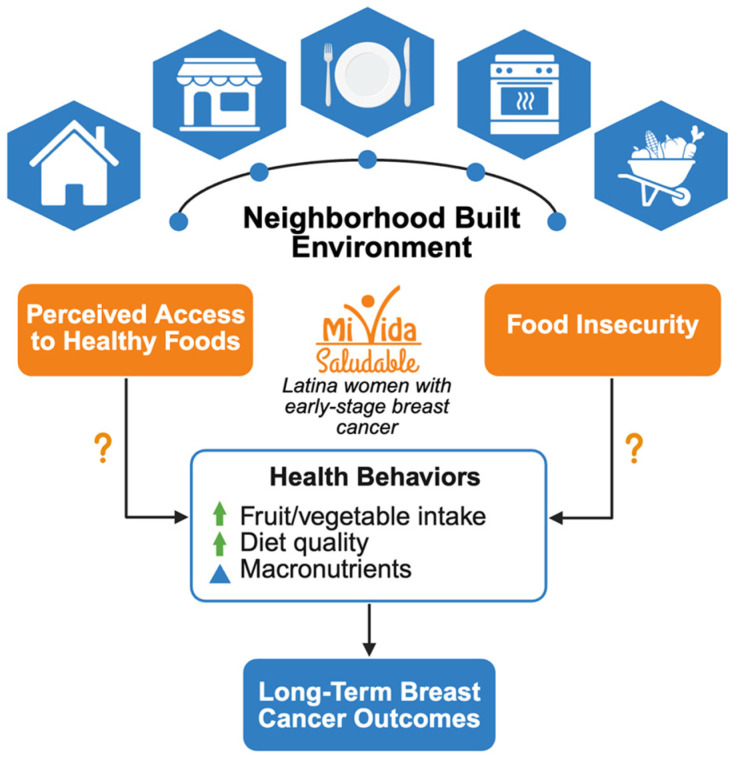
Conceptual model of the influence of the neighborhood built environment, perceived access to healthy foods, and food insecurity on health behaviors. Green arrow = increase in fruit/vegetable intake or diet quality. Blue triangle = change in macronutrient intake.

To address these research gaps, a secondary analysis of data from the *¡Mi Vida Saludable!* (MiVS) trial was conducted. The analysis aims to examine the associations between participants’ perception of access to healthy foods (AHF) and food insecurity (FI) on participants’ fruit and vegetable (FV) intake, select dietary measures, diet quality, and body mass index (BMI) in a sample of urban-dwelling Hispanic/Latina breast cancer survivors (Figure 1). The main outcomes manuscript for MiVS, which was accepted at the time of this publication, found that FV intake only increased in the in-person session trial arm [20,21].

## 2. Materials and Methods

### 2.1. Study Population

The *¡Mi Vida Saludable!* (MiVS) study was a 12-month, 2 × 2 factorial randomized-controlled trial in Latina breast cancer survivors (*n* = 167) living in New York City [21]. The goal of the MiVS study was to test the separate and joint effects of lifestyle interventions, which consisted of in-person group education sessions and eHealth (supportive and educational text messaging) components, on FV intake and physical activity. The eligibility criteria for the MiVS study were being female, self-identifying as Latina/Hispanic, being aged 18 years or older, having a history of early-stage BC, being over three months post-treatment, having no evidence of metastatic or recurrent disease, and consuming less than five daily servings of FV and/or engaging in less than 150 min per week of MVPA. All study participants provided written informed consent, and the study was approved by Institutional Review Boards from Columbia University Irving Medical Center in New York City, New York, and the Fred Hutchinson Cancer Center in Seattle, Washington. The study protocol and participant baseline characteristics have been published [21,22].

### 2.2. Study Design

Eligible participants were randomized into one of four study arms: (1) four weekly in-person group sessions (in-person component) and 11 months of eHealth communications (eHealth component); (2) in-person sessions only; (3) eHealth communications only; or (4) control group. The in-person weekly group sessions intervention involved participatory cooking classes, exercise sessions, and visits to local food markets. The eHealth communication intervention included weekly text messages with motivational content on diet and physical activity tailored for cancer survivors, bi-monthly email newsletters, and unlimited access to online content on nutrition for cancer survivors from the study website, “Cook for Your Life” (cookforyourlife.org). Summarized national guidelines on nutrition and physical activity from the American Institute for Cancer Research (AICR) and the American Cancer Society (ACS) were provided to all participants in written format and by staff verbally, including those women randomized to the control arm. All participants were also given a Fitbit Zip wearable device as a study incentive and to support self-monitoring of their physical activity. Additional details on the MiVS interventions, including the content development process and validation for the intervention, have been published [21,22].

### 2.3. Data Collection and Measures

Following eligibility screening, baseline data collection by study staff occurred in the following stages: (1) in-person clinic visit for completion of informed consent, medical records release, and study questionnaires; (2) three telephone-administered 24 h dietary recalls for dietary assessment; and (3) a final clinic visit for collection of anthropometric measurements, optional blood draw, and staff-administered 7-day physical activity recall. At baseline, all participants received an overview of diet, nutrition, and physical activity recommendations for cancer survivors by study staff and were provided with the Fitbit Zip device. Data collection procedures were repeated at 6 months and again at 12 months, with the 12-month collection also including an exit interview. Over a 33-month recruitment period (January 2016 to September 2018), data collection was conducted in five waves of participant cohorts.

The first two cohorts of study participants indicated that the baseline questionnaire was too burdensome and lengthy. Therefore, subsequent cohorts were given a shorter questionnaire that excluded questions on the scale used to measure perceived access to health foods (AHF). Thus, the sample for analyses of AHF is smaller (*n* = 86) than the sample for analyses of FI (*n* = 157).

The AHF questions characterized the participants’ perceived neighborhood food environment and were modified from a previously validated scale capturing self-reported neighborhood characteristics in urban Latino/Hispanic and African American populations [23]. Participants reported their agreement using a 5-option Likert scale to the following statements: (1) A large selection of fresh fruits and vegetables is available in my neighborhood; (2) The fresh fruits and vegetables in my neighborhood are of high quality; (3) A large selection of low-fat products is available in my neighborhood; and (4) There are many opportunities to purchase fast food in my neighborhood. Responses were summed (statement 4 was reverse scored), resulting in a score ranging from 4 to 20, with higher values reflecting a more favorable perception of having AHF in their neighborhood. Two groups characterized as low-to-medium (low–medium) and high access were created, based on scores of less than 14 or 14 or more, respectively. The cut-point value of 14 was selected to ensure a relatively even distribution of respondents in both groups and to assure those in the “high” group reported most statements favorably.

The Six-Item Short Form of the Food Security Survey Model, a modified short form of the “Household Food Security Scale”, was used to assess FI [24]. Participants were asked three questions from this modified short form, considered a reliable approach to minimize respondent burden [25,26]. Participants were defined as food insecure if they provided a “yes” response to any of the following questions: (1) In the past 12 months, did (you/you or other adults in your household) ever cut the size of your meals or skip meals because there wasn’t enough money for food?; (2) In the past 12 months, did you ever eat less than you felt you should because there wasn’t enough money for food?; (3) In the past 12 months, were you ever hungry but didn’t eat because there wasn’t enough money for food?

Dietary intake was measured from three 24 h dietary recalls administered at baseline, 6 months, and 12 months. Dietary intake data were collected and analyzed using Nutrition Data System for Research (NDSR) software versions 2016–2019, developed by the Nutrition Coordinating Center (NCC), University of Minnesota, Minneapolis, MN [27,28,29]. For this analysis, specific dietary measures including FV intake, energy density, total calories, total protein, total sugars, total dietary fiber, and total fat were used. Total fat was evaluated by subtypes, such as trans-saturated fats and monounsaturated and polyunsaturated fatty acids. Information about sugar intake referenced foods containing added sugars, such as sugar-sweetened beverages (soda, energy drinks, and fruit drinks), desserts, baked goods, breakfast cereals, flavored yogurts, and condiments like ketchup and barbecue sauce; it did not include naturally occurring sugars in foods like whole fruits. Diet quality was based on scores of the Healthy Eating Index 2015 (HEI-2015), which ranged from 0 to 100, with higher scores suggesting better diet quality [30].

Baseline questionnaires collected demographic and socioeconomic characteristics, which were age, marital status, highest education level achieved, annual household income, home ownership, housing type, number of people in the household, and employment status. Cultural factors were nationality, acculturation, and whether US-born or foreign-born. Acculturation was based on the Short Acculturation Scale for Hispanics (SASH), which has subscales for language and social relationships [31]. Scores ranged from 0 to 5, with higher scores reflecting being more accultured [31]. Participants also reported on their prevalence of 12 select comorbidities, which was summed up so that higher values indicated greater comorbidity [21]. Finally, study staff collected anthropometric measurements (weight, height, waist circumference, hip circumference, and waist-to-hip ratio) during all clinic visits. The measures of weight, in kilograms, and height, in meters, were used to calculate BMI.

### 2.4. Statistical Analyses

Baseline demographic, socioeconomic, and clinical characteristics were described using means and standard deviations (SD) for continuous variables and frequencies for categorical variables. Statistically significant differences between low–medium and high AHF and FI status were identified using *t*-tests for continuous variables and Chi-square tests for categorical variables. Adjusted means and SD for the diet measures, diet quality, weight, and BMI at baseline were estimated using analysis of variance, with adjustment for age and acculturation score in the analysis for AHF and adjustment for marital status and home ownership in the analysis for FI.

Outcomes were evaluated for normality in their distributions. Due to concerns about normality of residuals for FV intake, this outcome measure was log-transformed and percent change from baseline was reported. Generalized estimating equations (GEE) using the identity link function and an exchangeable working correlation matrix were fit to estimate the association of AHF and FI on diet and BMI. These models provided estimates of the changes in the diet and BMI measures from baseline to six months and baseline to 12 months for study participants with low–medium AHF, high AHF, and FI status. Interaction terms between time and the exposure variables (AHF and FI) were included to identify statistically significant differences between the exposure groups. All models adjusted for baseline age, marital status, education level, and study arm to address potential confounding.

To determine whether there were intervention effects on these associations, we included adjustment for study arm in the GEE model as part of a sensitivity analysis. These statistical models included a 3-way interaction term for time, arm, and exposure (AHF or FI) to determine if there was a statistically significantly different 12-month change among high AHF (or food secure) relative to low–medium AHF (or food insecure) for women randomized to an intervention compared to those in the control arm.

To address AHF and FI as potential overlapping constructs, we generated cross-tabulations and estimated Spearman correlations. Statistical significance was based on an alpha of 0.05 and analyses were executed with SAS statistical software version 9.4 (SAS Institute, Cary, NC, USA).

## 3. Results

AHF was collected in 86 of 167 MiVS study participants, with 58% perceiving low–medium AHF and 42% perceiving high AHF. FI was measured in 157 of 167 participants and was prevalent in 23% of women. Baseline demographic and socioeconomic characteristics by AHF and FI are presented in Table 1. Women who perceived to have high AHF were more likely to be older (59 vs. 53 years, *p* = 0.002) and had lower overall SASH scores (1.8 vs. 2.1, *p* = 0.01). Women with FI were more likely to be single (44% vs. 16%, *p* = 0.003) and renting their home (100% vs. 86%, *p* = 0.02). Among women with FI, 60% had a household income between $0–$15,000 per year, while 53% of women without FI had a household income between $0–$15,000 per year (*p* = 0.31). The most common self-reported nationality of participants was Dominican (70%), followed by Puerto Rican (10%), Ecuadorian (6%), Colombian (4%), Mexican (3%), and other countries (7%).

Baseline diet measures, weight, and BMI by AHF and FI are shown in Table 2. Women with low–medium AHF had lower mean protein intake compared to women with high AHF (45 vs. 53 g/day, *p* = 0.04); no other significant differences in the baseline outcome measures were observed. Women with FI had higher mean weight (78 vs. 72 kg, *p* = 0.03), and higher BMI (31 vs. 29 kg/m^2^, *p* = 0.02), compared to women without FI. No differences were noted in any diet measures when comparing women with versus without FI.

Women with low–medium AHF had a 19% decrease (95% CI: −41%, +12%) in daily FV intake from baseline to six months and a 32% decrease (95% CI: −51%, −7%) in daily FV servings from baseline to 12 months (Table 3). In contrast, women with high AHF had 3% (95% CI: −25%, +26%) and 17% (95% CI: −40%, +13%) decreases in FV intake at 6 and 12 months, respectively; however, between-group differences were not statistically significant. Women with low–medium AHF had a small reduction in energy density from baseline to 12 months (−0.05 kcal/g, 95% CI: −0.11, 0.01). However, women with high AHF had a 0.06 kcal/g (95% CI: 0.00, 0.12) increase in energy density at 12 months, and the between-group difference was statistically significant (*p* ≤ 0.05). Additional statistically significant baseline to 12-month decreases were observed for women with low–medium AHF, including for total caloric intake (−129.0 kcal, 95% CI: −244.5, −13.4), total carbohydrates (−26.7 g, 95% CI: −45.0, −8.5), total sugars (−16.8 g, 95% CI: −26.1, −7.5), and total fat (−5.8 g, 95% CI: −10.6, −1.1); BMI decreased though not significantly (Table 3). Women with high AHF had marginal (not statistically significant) increases in total caloric intake, total carbohydrates, total sugars, and BMI, and a statistically significant increase in energy density at 12 months (0.06 kcal/g, 95% CI: 0.003, 0.12). In contrast, women with low–medium AHF had small increases in total protein and Healthy Eating Index total scores, while women with high AHF had small decreases in both these measures though these changes were not statistically significant.

For the between-group comparisons of AHF, women with low–medium AHF had lower energy density, total carbohydrates, total sugars, and BMI at 12 months compared to baseline; these changes were statistically significantly different (all *p* ≤ 0.05) from the changes observed in women with high AHF (Table 3). No statistically significant between-group differences were observed for changes in total protein and Healthy Eating Index.

**Figure 2:**
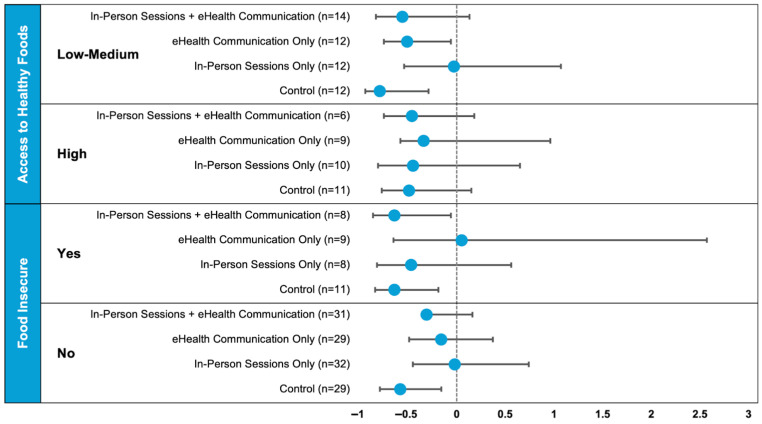
Percent change in fruit–vegetable intake from baseline to 12 months by Access to Healthy Foods and Food Insecurity Stratified by Study Arm. Analyses adjusted for baseline age, marital status, and education. Data were log-transformed; therefore, estimates, confidence intervals, and *p*-values relate to the percent change and ratio of percent change.

Women with FI had a 30% reduction (95% CI: −50%, −4%) and 39% reduction (95% CI: −57%, −14%) in FV servings per day from baseline to 6 and 12 months, respectively (Table 3). Women with FI had a significant reduction in total sugar intake at 12 months (−11.7 g, 95% CI: −23.2, −0.3). Women without FI had a 2% increase (95% CI: −14%, +20%) in daily FV intake from baseline to 6 months and a 10% decrease (95% CI: −25%, +8%) from baseline to 12 months, though these changes were not statistically significant. Women without FI had statistically significant reductions in total fat intake at both 6 and 12 months: −3.9 g (95% CI: −7.2, −0.7) and −4.8 g (95% CI: −8.6, −1.0), respectively. Women without FI had a statistically significant reduction in BMI at 6 months (−0.24 kg/m^2^, 95% CI: −0.48, −0.01); however, this change did not persist at 12 months. For the other outcome measures, in both FI groups, marginal decreases were observed in most diet measures and in BMI, with a trend toward improvement in the Healthy Eating Index, although the changes in these other measures were not statistically significant. The differences in changes in FV servings were statistically significantly different between FI groups at both 6 and 12 months (*p* ≤ 0.05). Decreases in total fat, total protein, total caloric intake, total carbohydrates, and total sugars tended to be larger in the women with FI, but no between-group comparisons were statistically significant besides FV servings. The percent change in FV servings consumed per day from baseline to 12 months by AHF and FI group stratified by the MiVS intervention study arm is depicted in Figure 2. Consistent with the results reported for the full sample, daily FV intake generally decreased over the 12-month period. Statistically significant within-group reductions in FV intake from baseline to 12 months were seen for the following groups: women with low–medium AHF who were randomized to the eHealth communication only or the control arms; for women with FI who were randomized to the in-person plus eHealth communication or control arms; and for women without FI who were randomized to the control arm.

Among women with both AHF and FI data (*n* = 85), 33% of the 21 women with FI also reported high AHF (*p* = 0.34). Of the 64 women who did not have FI, 45% reported high AHF. Moreover, no significant correlations between FI and AHF were noted (r= −0.105, *p* = 0.34).

## 4. Discussion

### 4.1. General Discussion

In this analysis of Hispanic/Latina women with a history of early-stage breast cancer, on a clinical trial of a lifestyle intervention aiming to increase fruit and vegetable intake and physical activity, perceived AHF in their neighborhoods and FI were associated with dietary changes over 12 months. Women reporting low–medium AHF had statistically significant reductions in total caloric intake, carbohydrates, sugars, and fat, while women who reported high AHF did not. Yet, women with lower perceived AHF also had significant reductions in FV intake over 12 months, while women with perceived higher AHF did not. Similarly, daily FV intake reductions were larger among women with FI than among women without. Stratification by the MiVS study intervention arms, and AHF and FI, showed that FV intake decreased for participants over 12 months, particularly among women with perceived low–medium AHF or FI, with the largest reductions observed for women in the control group. In the main outcomes analysis, reported in the main outcomes paper (accepted for publication at the time of this publication), which was not stratified by AHF or FI, FV intake increased in the in-person-only group.

The American Cancer Society (ACS) highlights the importance of addressing FI and lack of AHF as barriers to meeting their recommended nutrition and physical activity guidelines for cancer survivors [19]. The ACS, the Behavioral Risk Factors Surveillance System (BRFSS), and a sample of underserved ethnic minority cancer survivors in New York City further note substantial FI differences among cancer survivors according to race and ethnicity, with Hispanic and Black people having a greater risk for FI compared to non-Hispanic White people [32,33]. Our findings emphasize the importance of integrating both food environment and FI measures into the design of dietary interventions, particularly in populations with specific food access considerations.

While this analysis may be the first to examine associations between perceived food environment and dietary outcomes in BC survivors, these findings are consistent with studies performed in other populations. For example, a systematic review of 19 studies, 13 of which were performed in the US, found that having a more positive perception of one’s food environment was generally associated with higher intake of healthier foods, although this was not consistent across all studies [34]. In this review, Yamaguchi et al. distinguished five dimensions of perceived “food access”, specifically accessibility, availability, affordability, acceptability, and accommodation [35,36], and reported that dietary intakes of healthy or unhealthy foods varied across each dimension [34]. In our analysis using MiVS data, the scale we used captured dimensions of availability (Statements 1, 3, and 4 from Methods) and acceptability (Statement 2), but we estimated a summary score that was intended to measure the overall food environment. A lack of precision in distinguishing the food environment dimensions likely contributed to our inconsistent findings. More exploration across these dimensions is needed.

A similar exploration was conducted using data from our *¡Cocinar Para Su Salud*! (Cook For Your Health!) study, which predated and provided pilot data for the MiVS study. In the *¡Cocinar Para Su Salud*! (Cook For Your Health!) study, Feathers et al. demonstrated that objective measures of neighborhood-level access to produce were linked to both trial participation and increased FV consumption among Latina breast cancer survivors in New York City, although these were not statistically significant trends [37]. However, a study by Janda and colleagues in a cohort of predominately urban-dwelling Hispanic people in Central Texas, 40% of whom experienced food insecurity, found that proximity to a supermarket was independently associated with greater FV consumption [38]. Furthermore, evidence suggests that the overall relationship between perceived and objective neighborhood food environment measures does not correlate, as reported by Lucan et al. in urban Pennsylvanians [39]. Indeed, these examples specifically focus on accessibility as it relates to distance and proximity to locations where food is sold and therefore represents a different dimension of the food environment than those we analyzed for this study. Taken together, subjective and objective measures capture distinct aspects of the food environment, and both should be collected in future work to provide a more comprehensive perspective.

Among women with food insecurity, our results showed statistically significant decreases in FV intake at 6 and 12 months and these changes were significantly different compared to those observed women not experiencing food insecurity. The cross-sectional study by Janda and colleagues cited above also reported associations between food insecurity and FV consumption in a mostly urban-dwelling population without cancer, noting lower FV consumption among food insecure non-Hispanic Whites compared to food secure non-Hispanic Whites [34]; however, the authors also noted a significant interaction between FV consumption, food insecurity, and race/ethnicity, where food insecure Hispanics had higher FV consumption compared to food secure non-Hispanic Whites. In our cohort, 23% of the women were food insecure; this is similar to findings from a 2025 US Preventive Services Task Force Evidence Report and Systematic Review that reported the prevalence of food insecurity to be 20.8% among Hispanic households, compared to 9.3% among White households [40]. In one fair-quality randomized crossover study highlighted in the review, provision of home-delivered, medically tailored meals to people with diabetes reduced food insecurity and may be a strategy to consider testing in future prospective trials in BC survivors [41].

Our findings showed women with FI had significantly less protein intake at 6 months compared to baseline; however, this decrease in protein intake was not significant at 12 months and was not significant between the two groups. Protein is an important nutrient for BC survivors. In the Nurses’ Health study, higher protein intake among women with stages I-III BC was associated with improved survival and reduced risk of distance recurrence [42]. The ACS and National Comprehensive Cancer Network recommend BC survivors obtain protein from a variety of sources including animal-based proteins low in saturated fat (e.g., fish, lean poultry, eggs, low-fat dairy) and plant-based proteins (e.g., nuts, seeds, and legumes), including up to three servings of whole-soy foods daily (References). Given the trend toward reduced protein intake among women with FI in our study, and its importance for BC survivors, future trials should aim to capture total protein intakes and types of protein sources in BC survivors.

Our results also revealed paradoxical reductions in calorie and nutrient intake alongside small declines in BMI in women with FI or lower AHF. These differences may not truly reflect a healthier pattern of eating, but rather constrained intake due to limited resources [34,35,43]. For example, Zenk and others highlight that BMI changes in a multiethnic urban population were related to the size of grocery stores and perceptions of FV availability [44,45,46]. While limitations of this study (e.g., small sample size and scope of the data collected) prohibit a similar examination using MiVS data, ongoing research exploring the intersections of perceived and objective food access with diet quality and weight trajectories in this population is recommended [44,45,46]. Furthermore, exploration of changes in body composition, namely fat mass and lean mass, could be helpful in elucidating whether the women with FI or lower AHF in our study engaged in a healthier diet. However, objective measures of body composition using methods such as dual-energy X-ray absorptiometry scans or bioelectrical impedance analysis were not collected in this clinical trial. While body circumferences, including waist and hip were collected, evidence has shown that body composition formulas using circumference measures tend to be biased in race and ethnic groups of color (e.g., not non-Hispanic White) [47,48]. Thus, interpretation of any findings using these formulas may be incorrect or misleading.

Nativity and acculturation are other factors associated with food choices that are important to consider in Hispanic/Latina women with a history of BC. Results from the ENCLAVE study found that foreign-born women ate healthier diets compared to US-born women, including more fruits and vegetables, and less butter and red meat, in a diverse group of BC survivors including Hispanic/Latina and Asian women [49]. Additionally, foreign-born Hispanic/Latina BC survivors had lower BMI and smaller waist circumference compared to US-born counterparts in the ENCLAVE study [50]. Findings in MiVS were similar: women with higher acculturation to US culture had higher waist circumference, poorer diet quality, and higher fat intake compared to women with lower acculturation scores, which might suggest the latter group is retaining dietary preferences more aligned with their birth country [20]. Furthermore, our analysis showed women with higher perceived AHF had lower acculturation scores than women with perceived low AHF. Taken together, these data support continued collection of data on nativity and acculturation among Latina/Hispanic women to account for relationships between food choices and anthropometric measures relevant to BC survivors.

### 4.2. Strengths and Limitations

This analysis is among the few to report on food insecurity and access to healthy food among urban-dwelling Latina breast cancer survivors. The use of validated measures of food insecurity and nutritional intake enhances the rigor and comparability of our study. There are also limitations to our analysis. First, the prevalence of FI was low in our population (23%), limiting the power of between-group comparisons. Second, the length of the initial questionnaires introduced participant burden. The AHF questionnaire was subsequently removed from the baseline intake, which limited data availability and statistical power to assess changes over time and compare groups. Finally, the AHF portion of the questionnaire did not include all the questions from the original questionnaire, which potentially limits its reliability in capturing this measure.

### 4.3. Future Directions

Future investigators are encouraged to consider food insecurity and access to healthy foods (both objective and perceived measures) in study design and recruitment strategies. Additional data in this area of research can help inform future nutrition trials that aim to improve FV intake among breast cancer survivors who are likely to experience food insecurity and low access to healthy food, like the urban-dwelling Latina breast cancer survivors in our study. Furthermore, our results suggest women with low–medium AHF had healthier macronutrient profiles, and it is unclear whether this resulted from intentional healthy eating choices or resource constraints. Future studies should consider including measures that capture people’s motivations behind their food choices to differentiate between conscious healthy food choices, food environments, and resource availability.

## 5. Conclusions

Over half of the Latina BC survivors in New York City who participated in a 12-month diet and PA intervention trial had low–medium AHF at baseline; nearly one quarter had FI. Women with low to medium AHF and women with FI had significant reductions in FV intake over 12 months, while women with high AHF and without FI did not. In the analysis stratified by trial arm, women in the control arm, with low–medium AHF and FI, had larger decreases in FV intake after 12 months than women in the intervention arms, although sample sizes across strata were small. Future dietary intervention studies in Latina BC survivors, and other underserved or under-resourced populations, should account for AHF and FI in the study design, and develop and test strategies to connect people to healthy food resources.

## Figures and Tables

**Table 1:** Baseline characteristics of Latina breast cancer survivors in *¡Mi Vida Saludable!* by access to healthy foods and food insecurity.

	Access to Healthy Foods (*n* = 86)			Food Insecure (*n* = 157)		
Characteristic	Low–Medium	High	*p*	Yes	No	*p*
N (%)	50 (58)	36 (42)		36 (23)	121 (77)	
Randomization arm, n (%)			0.66			0.85
In-person sessions + eHealth communication	14 (28)	6 (17)		8 (22)	31 (26)	
eHealth communication only	12 (24)	9 (25)		9 (25)	29 (24)	
In-person sessions only	12 (24)	10 (28)		8 (22)	32 (27)	
Control	12 (24)	11 (31)		11 (31)	29 (24)	
Age, years, mean (SD)	**53.2 (8.6)**	**59.4 (9.6)**	**<0.01**	56.5 (7.9)	57.0 (10.7)	0.79
Education level, n (%)			0.43			0.24
High school graduate/GED or less	21 (42)	19 (53)		21 (58)	53 (44)	
Some college	15 (30)	11 (31)		9 (25)	33 (27)	
College graduate or more	14 (28)	6 (17)		6 (17)	35 (29)	
Annual household income, n (%)			0.84			0.31
$0–$15,000	26 (55)	22 (61)		21 (60)	63 (53)	
$15,001–$30,000	10 (21)	6 (17)		8 (23)	21 (18)	
More than $30,000	11 (23)	8 (22)		6 (17)	36 (30)	
Has paid employment (full or part-time), n (%)	20 (41)	12 (33)	0.48	14 (40)	47 (39)	0.90
Marital status, n (%)			0.76			**<0.01**
Married/living with partner/common law	18 (36)	12 (33)		8 (22)	53 (44)	
Separated/divorced	19 (38)	13 (36)		10 (28)	43 (36)	
Widowed	2 (4)	3 (8)		2 (6)	6 (5)	
Single	11 (22)	8 (22)		16 (44)	19 (16)	
Participates in EBT/SNAP or WIC, n (%)	29 (58)	19 (53)	0.63	22 (61)	58 (48)	0.17
Is food insecure, n (%)	14 (29)	7 (19)	0.34	---	---	---
Number in household, mean (SD)	2.9 (1.3)	2.9 (2.1)	0.92	2.9 (1.7)	2.9 (1.5)	0.88
Housing type, n (%)						0.11
Apartment	46 (92)	32 (89)		29 (83)	106 (88)	
House	2 (4)	4 (11)		2 (6)	13 (11)	
Single room occupancy	2 (4)	0		4 (11)	2 (2)	
Owns home, n (%)	2 (4)	5 (14)	0.11	**0**	**16 (14)**	**0.02**
US born, n (%)	7 (14)	2 (6)	0.21	6 (17)	25 (21)	0.60
SASH acculturation scores (0–5), mean (SD)						
Language	**1.8 (0.72)**	**1.5 (0.61)**	**0.02**	1.78 (1.08)	1.8 (0.9)	0.92
Social	2.3 (0.70)	2.1 (0.57)	0.17	2.27 (0.91)	2.2 (0.6)	0.73
Overall	**2.1 (0.57)**	**1.8 (0.48)**	**0.01**	2.04 (0.89)	2.0 (0.7)	0.90
Has high acculturation, n (%)	2 (4)	1 (3)	0.75	7 (19)	11 (9)	0.09
Comorbidity index, mean (SD)	1.7 (1.8)	1.6 (1.7)	0.96	1.9 (1.5)	1.4 (1.7)	0.11

EBT: electronic benefit transfer; SD: standard deviation; SASH: Short Acculturation Scale for Hispanics; SNAP: Supplemental Nutrition Assistance Program; WIC: Women, Infants, and Children supplemental nutrition program. Bold values indicate the difference between groups is a statistically significant difference at *p* ≤ 0.05.

**Table 2:** Baseline-adjusted diet and anthropometric measures in *¡Mi Vida Saludable!* by AHF and FI.

	Access to Healthy Foods * (*n* = 86)			Food Insecure ^†^ (*n* = 157)		
Diet Measures	Low–Medium	High	*p*	Yes	No	*p*
FV intake **^‡^**, servings/day	3.7 (0.5)	4.4 (0.6)	0.35	4.5 (0.5)	3.7 (0.3)	0.16
Energy density, kcal/g	0.64 (0.03)	0.61 (0.03)	0.51	0.68 (0.03)	0.62 (0.02)	0.14
Total caloric intake, kcal	1150 (51)	1173 (61)	0.78	1200 (74)	1192 (40)	0.93
Total carbohydrates, g	160.8 (8.1)	157.3 (9.6)	0.79	160.9 (9.8)	160.7 (5.3)	0.99
Total protein, g	**45.3 (2.2)**	**52.8 (2.6)**	**0.04**	52.6 (3.6)	51.2 (2.0)	0.75
Total sugars, g	62.0 (4.4)	63.4 (5.2)	0.85	65.5 (5.1)	62.6 (2.7)	0.61
Total dietary fiber, g	15.6 (1.0)	16.4 (1.2)	0.64	15.7 (1.3)	16.0 (0.7)	0.87
Total fat, g	38.9 (2.3)	38.2 (2.7)	0.84	39.8 (3.5)	40.7 (1.9)	0.83
Total monounsaturated fatty acids, g	14.9 (1.0)	15.5 (1.1)	0.68	14.6 (1.3)	15.6 (0.7)	0.51
Total polyunsaturated fatty acids, g	8.4 (0.6)	8.0 (0.7)	0.67	9.1 (0.9)	8.8 (0.5)	0.78
Total saturated fatty acids, g	12.3 (0.9)	11.4 (1.0)	0.53	12.5 (1.2)	12.6 (0.6)	0.97
Total trans-fatty acids, g	0.7 (0.1)	0.7 (0.1)	0.98	0.8 (0.1)	0.8 (0.1)	0.77
HEI 2015	70.4 (1.7)	72.4 (2.0)	0.46	66.5 (2.2)	70.5 (1.2)	0.11
**Anthropometric Measures**						
Weight, kg	74.8 (2.1)	73.3 (2.4)	0.66	**77.9 (2.5)**	**71.8 (1.3)**	**0.03**
BMI, kg/m^2^	29.8 (0.7)	29.7 (0.9)	0.93	**31.3 (0.9)**	**29.2 (0.5)**	**0.05**

BMI: body mass index; FV: fruit and vegetable; HEI: Healthy Eating Index * Adjusted for age and SASH (Short Acculturation Score for Hispanics) score; **^†^** Adjusted for marital status and home ownership; **^‡^** Excluded juices, potatoes, and dried fruits. Bold values indicate 6- and/or 12-month changes within groups is a statistically significant difference at *p* ≤ 0.05.

**Table 3:** Six and twelve-month change * from baseline in diet measures and BMI from baseline by AHF and FI.

	**Low–Medium AHF (*n* = 50)**		**High AHF (*n* = 36)**	
	**6-Month D**	**12-Month D**	**6-Month D**	**12-Month D**
	**Estimate (95% CI)**	**Estimate (95% CI)**	**Estimate (95% CI)**	**Estimate (95% CI)**
FV intake, servings/day **^†,‡^**	−19% (−41%, +12%)	**−32% (−51%, −7%)**	−3% (−25%, +26%)	−17% (−40%, +13%)
Energy density, kcal/g **^§^**	−0.002 (−0.08, 0.08)	−0.05 (−0.11, 0.01)	0.03 (−0.04, 0.10)	**0.06 (0.003, 0.12)**
Total caloric intake, kcal	−109.4 (−228.1, 9.3)	**−129.0 (−244.5, −13.4)**	15.9 (−96.8, 128.7)	13.1 (−107.2, 133.5)
Total carbohydrates, g **^§^**	−18.7 (−38.2, 0.8)	**−26.7 (−45.0, −8.5)**	5.8 (−11.2, 22.9)	6.8 (−11.4, 25.1)
Total protein, g	−0.2 (−5.5, 5.2)	5.2 (−0.2, 10.6)	−0.2 (−7.7, 7.4)	−0.8 (−8.4, 6.8)
Total sugars, g **^§^**	−7.5 (−17.5, 2.4)	**−16.8 (−26.1, −7.5)**	−3.1 (−12.7, 6.4)	1.0 (−9.8, 11.9)
Total fat, g	−4.7 (−9.4, 0.1)	**−5.8 (−10.6, −1.1)**	−0.2 (−5.5, 5.1)	−1.0 (−7.1, 5.1)
HEI 2015	−0.9 (−5.7, 3.8)	0.7 (−3.0, 4.4)	0.8 (−3.7, 5.3)	−3.3 (−8.0, 1.4)
BMI, kg/m **^†,§^**	−0.07 (−0.5, 0.3)	−0.3 (−0.7, 0.2)	**0.4 (0.1, 0.8)**	**0.3 (0.0, 0.6)**
	**Food Insecure (*n* = 36)**		**Not Food Insecure (*n* = 121)**	
	**6-Month D**	**12-Month D**	**6-Month D**	**12-Month D**
	**Estimate (95% CI)**	**Estimate (95% CI)**	**Estimate (95% CI)**	**Estimate (95% CI)**
FV intake, servings/day **^†,‡,§,||^**	**−30% (−50%, −4%)**	**−39% (−57%, −14%)**	2% (−14%, +20%)	−10% (−25%, +8%)
Energy density, kcal/g	0.01 (−0.09, 0.11)	−0.06 (−0.12, 0.01)	0.01 (−0.03, 0.05)	−0.02 (−0.05, 0.02)
Total caloric intake, kcal	−96.2 (−306.1, 113.7)	−120.9 (−279.9, 38.2)	−55.9 (−124.7, 13.0)	−44.3 (−120.7, 32.1)
Total carbohydrates, g	−4.0 (−35.8, 27.7)	−18.2 (−39.8, 3.4)	−5.3 (−15.2, 4.6)	−3.2 (−14.3, 7.9)
Total protein, g	**−8.4 (−15.7, −1.0)**	−3.8 (−11.3, 3.7)	−0.8 (−4.9, 3.3)	1.9 (−2.1, 5.9)
Total sugars, g	−1.2 (−19.0, 16.6)	**−11.7 (−23.2, −0.3)**	−3.0 (−8.0, 2.0)	−4.8 (−10.8, 1.1)
Total fat, g	−4.8 (−13.0, 3.5)	−4.0 (−11.0, 3.0)	**−3.9 (−7.2, −0.7)**	**−4.8 (−8.6, −1.0)**
HEI 2015	2.1 (−1.9, 6.1)	1.5 (−2.5, 5.5)	1.2 (−1.5, 3.9)	0.09 (−2.3, 2.5)
BMI, kg/m^2^	−0.19 (−0.73, 0.35)	−0.11 (−0.61, 0.39)	**−0.24 (−0.48, −0.01)**	−0.01 (−0.55, 0.54)

AHF: access to healthy foods; BMI: body mass index; FV: fruit–vegetable; HEI: Healthy Eating Index (2015) total score. * Adjusted for baseline age, marital status, education, and study arm; **^†^** Excluded juices, potatoes, and dried fruits; **^‡^** Data were log-transformed; therefore, estimates, 95% CIs, and *p*-values relate to the percent change; **^§^** 12-month changes between the two groups is statistically significantly different at *p* ≤ 0.05; **^||^** 6-month changes between the two groups is statistically significantly different at *p* ≤ 0.05. Bold values indicate 6- and/or 12-month changes within groups is a statistically significant difference at *p* ≤ 0.05. D indicates change over time.

## Data Availability

The datasets presented in this article are not readily available because the data are still being analyzed from the main study. Requests to access the datasets should be directed to Heather Greenlee.

## Abbreviations

The following abbreviations are used in this manuscript:

ACS	American Cancer Society
AHF	Access to healthy foods
AICR	American Institute for Cancer Research
BC	Breast cancer
BMI	Body mass index
BRFSS	Behavioral Risk Factor Surveillance System
FI	Food insecurity
FV	Fruit–vegetable intake
HEI-2015	Health Eating Index (2015)
MiVS	*¡Mi Vida Saludable*!
MVPA	Moderate-to-vigorous physical activity
NDSR	Nutrition Data System for Research
SASH	Short Acculturation Scale for Hispanics
SES	Socioeconomic status
US	United States
